# Antibiotic-specific differences in the response of *Staphylococcus aureus* to treatment with antimicrobials combined with manuka honey

**DOI:** 10.3389/fmicb.2014.00779

**Published:** 2015-01-27

**Authors:** Michael Liu, Jing Lu, Patrick Müller, Lynne Turnbull, Catherine M. Burke, Ralf C. Schlothauer, Dee A. Carter, Cynthia B. Whitchurch, Elizabeth J. Harry

**Affiliations:** ^1^The ithree institute, University of Technology, Sydney, Sydney, NSWAustralia; ^2^Comvita New Zealand Limited, Te PukeNew Zealand; ^3^School of Molecular Bioscience, University of Sydney, Sydney, NSWAustralia

**Keywords:** Medihoney, *Staphylococcus aureus*, manuka, antibiotic, synergy

## Abstract

Skin infections caused by antibiotic resistant *Staphylococcus aureus* are a significant health problem worldwide; often associated with high treatment cost and mortality rate. Complex natural products like New Zealand (NZ) manuka honey have been revisited and studied extensively as an alternative to antibiotics due to their potent broad-spectrum antimicrobial activity, and the inability to isolate honey-resistant *S. aureus*. Previous studies showing synergistic effects between manuka-type honeys and antibiotics have been demonstrated against the growth of one methicillin-resistant *S. aureus* (MRSA) strain. We have previously demonstrated strong synergistic activity between NZ manuka-type honey and rifampicin against growth and biofilm formation of multiple *S. arueus* strains. Here, we have expanded our investigation using multiple *S. aureus* strains and four different antibiotics commonly used to treat *S. aureus-*related skin infections: rifampicin, oxacillin, gentamicin, and clindamycin. Using checkerboard microdilution and agar diffusion assays with *S. aureus* strains including clinical isolates and MRSA we demonstrate that manuka-type honey combined with these four antibiotics frequently produces a synergistic effect. In some cases when synergism was not observed, there was a significant enhancement in antibiotic susceptibility. Some strains that were highly resistant to an antibiotic when present alone become sensitive to clinically achievable concentrations when combined with honey. However, not all of the *S. aureus* strains tested responded in the same way to these combinational treatments. Our findings support the use of NZ manuka-type honeys in clinical treatment against *S. aureus*-related infections and extend their potential use as an antibiotic adjuvant in combinational therapy. Our data also suggest that manuka-type honeys may not work as antibiotic adjuvants for all strains of *S. aureus*, and this may help determine the mechanistic processes behind honey synergy.

## INTRODUCTION

*Staphylococcus aureus* is a major causative agent of chronic wounds such as diabetic foot ulcers, venous leg ulcers, and pressure ulcers (Eady and Cove, 2003; Dowd et al., 2008; DeLeo and Chambers, 2009). These slow- or non-healing wounds pose a significant risk of sepsis and can result in invasive inflammatory disease such as infective endocarditis, which is associated with high mortality and morbidity (Orsi et al., 2002). In addition methicillin-resistant *S. aureus* (MRSA) strains have become resistant to most antibiotics both in the hospital and in the community (Zetola et al., 2005; Kardas-Sloma et al., 2011). One approach to combat the development of resistance is combination drug treatment (Greco et al., 1995). This improves treatment efficacy and enhances the value of existing antimicrobials in the absence of new drug development. In some cases, combinations of antimicrobials are synergistic, where the effect of two drugs in combination is significantly greater than the sum of each drug alone. This has the additional benefits of reducing both the treatment costs and the risk of possible side effects due to the lower concentrations of both agents used (Leibovici et al., 2010).

Naturally derived compounds like honey are gaining popularity as an alternative to antimicrobial compounds (Allen et al., 1991; Bogdanov et al., 2008). Honey is a natural product that has been applied to the topical treatment of infected chronic wounds (Molan and Cooper, 2000; Molan, 2006). Honey dressings and wound gels have been licensed by health authorities and are available to health professionals in many countries. Honey has a complex chemistry (Adams et al., 2008; Mavric et al., 2008), with established, broad-spectrum antibacterial activity against a diverse array of microorganisms, including those that are commonly associated with chronic wounds such as *S. aureus* and *Pseudomonas aeruginosa* (Blair et al., 2009; Henriques et al., 2010; Packer et al., 2012; Lu et al., 2013). NZ manuka honey, derived from nectar collected by honeybees (*Apis mellifera*) foraging on *Leptospermum scoparium*, is the major honey in clinical use today. Although the precise antimicrobial action of honey is unclear, several components have been identified that contribute toward its antimicrobial activity, including high sugar content, low water activity, low pH, and the formation of hydrogen peroxide upon dilution. In addition, methylglyoxal (MGO) has been identified as the dominant antimicrobial component of manuka honey (Adams et al., 2008; Mavric et al., 2008). Published clinical cases suggest that, in addition to killing infecting bacteria, medicinal manuka honeys promote chronic and acute wound healing by stimulating the host immune system (Gannabathula et al., 2012). However, honey represents a ‘challenge to the norm’ for healthcare workers and remains under-utilized in mainstream healthcare, often only used as a last line treatment when other therapies have failed. This is partly due to the lack of comprehensive scientific evidence supporting its clinical use (Cooper and Jenkins, 2012).

Bacteria appear unable to develop resistance to manuka honey, even when sub-inhibitory concentrations are used (Blair et al., 2009; Cooper et al., 2010). This is in contrast to antibiotics, where resistance is readily induced with sub-inhibitory exposure (Blair et al., 2009; Packer et al., 2012). This lack of resistance is probably due to the multiple antibacterial properties of honey that overwhelm bacterial stress responses (Blair et al., 2009; Jenkins et al., 2011, 2014). Manuka honey therefore offers a promising alternative for topical use, both as a single multi-component agent in its own right as well as in combination with antibiotics. Synergistic interactions between manuka honey and antibiotics, including oxacillin (Jenkins and Cooper, 2012b), tetracycline, imipenem, and mupirocin against the growth of a MRSA strain, EMRSA-15, have been reported (Jenkins and Cooper, 2012a). In our previous study we also found strong synergistic activity between manuka honey and rifampicin against multiple *S. aureus* strains, including clinical isolates and MRSA strains (Müller et al., 2013).

In this study we have expanded our investigation of honey synergy to include four antibiotics that are commonly administered to patients with staphylococcal infections: rifampicin, clindamycin, gentamicin, and oxacillin (Rayner and Munckhof, 2005), and have included a range of *S. aureus* strains including clinical isolates and MRSAs. Qualitative agar diffusion assays were performed, and checkerboard microdilution assays were used to determine if these combinations were quantitatively additive or synergistic against planktonic growth of *S. aureus*. The aims were: (1) to identify further novel honey-antibiotic therapies for staphylococcal infections; and (2) to determine whether antibiotic or strain-specific responses might occur. In addition, since biofilms are recognized to play a significant role in chronic wound infections (Percival et al., 2012), we tested whether synergy extends to the prevention of biofilm formation on an abiotic surface. We show that the combination of rifampicin and manuka honey yields the best result, being synergistic against all tested strains for both the inhibition of planktonic growth as well as the prevention of *S. aureus* biofilms. This was followed by the antibiotics clindamycin and oxacillin, which were synergistic with manuka honey for most strains, while additivity was observed with gentamycin and manuka honey. Responses were observed to be strain- and antibiotic-specific, indicating that synergy is not a generic process induced by honey, such as a general weakening of cells, but targets specific processes that may or may not enhance antimicrobial action. Our results support the use of manuka honey in combinational therapy of chronic wounds with antibiotics, and argues for a wider acceptance of honey in mainstream medicine.

## MATERIALS AND METHODS

### BACTERIAL STRAINS, MEDIA, AND ANTIMICROBIAL AGENTS

*Staphylococcus aureus* isolates included laboratory strain NCTC8325 and clinical isolates 04-227-3567 (non-MRSA), MW2 (MRSA; also known as USA400 and CA-MRSA; kindly provided by Dr. Barry Kreiswirth, Public Health Research Institute Center, New Jersey Medical School-Rutgers, The State University of New Jersey, USA) and RPAH18 (designated AUS-2 multi-resistance MRSA strain; and kindly provided by Dr. Jon Iredell, Westmead Hospital, Sydney, NSW, Australia). All planktonic growth and biofilm prevention assays were carried out using tryptone soya broth (TSB; Oxoid). Agar diffusion tests used TSB agar (TSB + 1% agar; Sigma–Aldrich) in 90 × 15 mm petri dishes. Antibiotics (rifampicin, oxacillin sodium salt, clindamycin hydrochloride, and gentamicin sulfate solution) were purchased from Sigma–Aldrich.

Two manuka-type honeys were used in this study: (1) unprocessed manuka honey sourced from *L. scoparium* plantations in Hokianga, New Zealand (MGO: 958 mg/kg; H_2_O_2_: 0.34 μmol/h); and (2) commercially available manuka honey (*L. scoparium* + *Kunzea ericoides*) in a proprietary formulation (Medihoney, MGO: 776 mg/kg; H_2_O_2_: 0.31 μmol/h). Both were provided by Comvita Ltd, New Zealand and were stored in the dark at 4°C. The concentrations of two major antimicrobial components in these NZ honeys, MGO, and hydrogen peroxide (as a production rate) were tested for this study and are equivalent to previously reported levels (Lu et al., 2014). In brief, MGO levels were analyzed against di-hydroxyacetone and expressed as milligram (mg) of MGO per kilogram (kg) of honey. The rate of production of H_2_O_2_ levels is expressed as micromole per hour (μmol/h) in 1 ml of 10% honey. Both honeys were diluted fresh for use in every assay. Honey concentrations are reported in this study as % weight/volume (w/v). A sugar solution comprising 45% glucose, 48% fructose, and 1% sucrose (w/v) was made to be isotonic with honey, and was also used to examine the effect of sugar alone or in combination with the antibiotics.

### DETERMINATION OF THE MINIMUM INHIBITORY CONCENTRATION (MIC) OF ANTIMICROBIAL AGENTS

Microdilution growth assays were used to assess the minimum inhibitory concentration (MIC) of antibiotics and honey against *S. aureus* strains. Routine static growth conditions were used against *S. aureus* clinical isolates 04-227-3567, MW2, and RPAH18 according to Clinical and Laboratory Standards Institute (CLSI) recommendations (CLSI, 2012), while shaking culture conditions were used for *S. aureus* strain NCTC8325 as its very strong tendency to form biofilms on abiotic surfaces under static growth conditions limited detection of planktonic growth in the liquid media phase. For both assays, diluted overnight bacterial culture (10^7^ CFU/mL, determined by CFU counting) was used to inoculate wells of a sterile 96-well flat-bottomed plate. Various concentrations of honeys or antibiotics were added to the designated wells by twofold serial dilutions with TSB growth media to a final volume of 150 μL. Untreated controls were also included. In the static growth assay, the plates were briefly shaken to mix the contents of each well and the optical density (OD) of each well was measured at 590 nm in a Synergy HT BioTek plate reader (BioTek Instruments Inc., USA). The plate was then incubated without shaking in a 37°C in a humidified incubator for 24 h, and the OD of each well was measured again at the end of incubation. The OD difference between the two time points was used to measure cell growth. In the shaking culture assay the plate was incubated in the same microtiter plate reader at 37°C with continuous moderate shaking to prevent biofilm formation (1800 rpm, amp. 0.549 mm x-axis) for 24 h, and was programmed to measure the OD hourly at 595 nm (Gen5 software, BioTek Instruments Inc., USA). For both growth assays, the MIC was defined as the lowest concentration of antimicrobial agent that inhibited 99% growth of *S. aureus* when compared to the untreated control.

### AGAR DIFFUSION TESTS TO ASSESS ANTIBIOTIC-HONEY INTERACTION AGAINST *S. aureus*

Fifty microliter aliquots of 10^9^ CFU/mL overnight culture of each of the *S. aureus* strains were spread uniformly onto TSB agar plates with or without 5% honey. This was previously determined to be a non-lethal, sub-inhibitory concentration of honey under these conditions (Müller et al., 2013). Paper disks impregnated with 4 μg of each antibiotic were then placed onto the agar surface. Inhibition zones were measured after 24 h incubation at 37°C. Assays were performed three separate times in duplicate. To determine the effect of 5% honey alone on bacterial growth, 20 μL of overnight culture (1 × 10^9^ CFU/mL) was diluted in 180 μL of PBS, followed by further serial dilutions (10^-1^–10^-8^). 20 μL of each dilution was then spotted onto a freshly prepared TSA plate with or without 5% honey (in triplicate). Colonies were counted after 24 h incubation at 37°C and CFUs were determined. As a control for the effect of sugar on inhibition of cell growth, a sugar solution made to be isotonic with honey was also used at 5% in combination with antibiotics.

Differences among the treatments (e.g., antibiotic alone, manuka honey-antibiotic, Medihoney-antibiotic and sugar solution-antibiotic) were assessed by one-way analysis of variance (ANOVA) with significance set at *p* ≤ 0.05. Dunnett’s test was performed *post hoc* for all assays where *p* < 0.05. This test further determines which of the three mean values of combination treatments (e.g., manuka, Medihoney, or sugar solution) is different from the antibiotic treatment alone. Statistical analyses were performed using GraphPad Prism ver.5.0c (Graphpad Software, San Diego, CA, USA).

### DETERMINATION OF MINIMUM BIOFILM INHIBITORY CONCENTRATION (MBIC) OF ANTIMICROBIAL AGENTS

Minimum biofilm inhibitory concentrations (MBICs) for the honey and antibiotics against *S. aureus* were determined using microdilution assays with crystal violet staining. Plates were prepared as for the MIC assays. Following incubation, the liquid from each well was carefully removed with a pipette and the wells were washed gently by rinsing with phosphate buffered saline (PBS) twice to remove loosely attached planktonic cells. The remaining biofilms were dry-fixed onto the plates by air-drying at 65°C for 1 h and then stained with 0.2% crystal violet solution for 1 h at room temperature. The excess crystal violet solution was decanted and the plates were rinsed with sterile reverse osmosis (RO) water, briefly dried, and the stained biofilms in each well were solubilized with 30% acetic acid. The OD of each well was measured at 595 nm using an automated Vmax plate reader (Molecular Devices, USA). OD readings were normalized to the untreated biofilm biomass and expressed as a percentage. MBICs were defined as the lowest concentration of antimicrobial agent that completely prevented the establishment of a biofilm (0% biofilm biomass) in the microtiter-plate well compared to the untreated control.

For checkerboard microdilution assays, each pair of antimicrobial agents (honey and antibiotic) were added across the *x* and *y* dimensions of a 96-well plate by twofold serial dilution with TSB growth media. Concentrations ranged from 0.03 to 1 × MIC for each antibiotic and from 2 to 32% for each honey. Each combination was repeated in the adjacent horizontal wells to provide technical duplicates. An overnight culture of *S. aureus* was then added to each well to give a final inoculum of approximately 10^7^ CFU/mL. Crystal violet staining of adherent biofilm was performed as described above. All checkerboard microdilution assays were repeated two times on two different days to provide experimental replicates.

Synergy was assessed using the fractional inhibition concentration index (FICI). For inhibition of planktonic growth, FICI = (MIC of antibiotic in combination/MIC of antibiotic alone) + (MIC of honey in combination/MIC of honey alone; Eliopoulos and Moellering, 1996); FICI for the inhibition of biofilm formation was calculated similarly, with MBIC replacing MIC in the formula. Synergy was defined as FICI ≤ 0.5, no interaction was defined as FICI > 0.5–4, and antagonism was defined as FICI > 4 (Odds, 2003).

## RESULTS

### MINIMUM INHIBITORY CONCENTRATIONS OF HONEYS AND ANTIBIOTICS AGAINST *S. aureus* STRAINS DURING PLANKTONIC GROWTH

The *in vitro* antibacterial activity against planktonic growth for Medihoney, manuka honey, and for each of the antibiotics was established by determining the MICs against the various *S. aureus* strains. The results are summarized in **Table 1**.

**Table 1:** Minimum inhibitory concentrations (MICs) of antimicrobial agents against *Staphylococcus aureus* strains.

**Strains**	**Minimum inhibitory concentrations**					
	**Manuka honey % (w/v)**	**Medihoney % (w/v)**	**Rifampicin (μg/ml)**	**Clindamycin (μg/ml)**	**Gentamicin (μg/ml)**	**Oxacillin (μg/ml)**
NCTC8325	8	8	0.04	0.3	0.625	0.25
RPAH18^1,2^	8	8	0.08	>20	>20	>20
MW2^1,2^	8	8	0.04	0.3	0.6	>20
04 227–3567^2^	8	8	0.04	0.3	0.5	0.25

*^1^MRSA strain;*

*^2^Clinical isolate*.

For both manuka honey and Medihoney, the MICs for all strains were 8% (w/v) and are consistent with our previous studies (Blair et al., 2009; Lu et al., 2013, 2014; Müller et al., 2013). The non-MRSA strains (NCTC8325 and 04 227-3567) had similar MICs for all four antibiotics (rifampicin, oxacillin, clindamycin, and gentamicin), which ranged from 0.04 to 0.625 μg/mL. MRSA strains (RPAH18 and MW2) displayed a more resistant profile, with RPAH18 being sensitive to only one of the four antibiotics (rifampicin) and MW2 sensitive to three (rifampicin, clindamycin, and gentamicin). These antibiotic MICs are consistent with those reported in the literature for *S. aureus* (Porthouse et al., 1976; Maduri Traczewski et al., 1983; Smith et al., 2009; Bauer et al., 2013).

### MOST HONEY–ANTIBIOTIC COMBINATIONS INTERACT POSITIVELY TO INHIBIT THE GROWTH OF *S. aureus*

**FIGURE 1:**
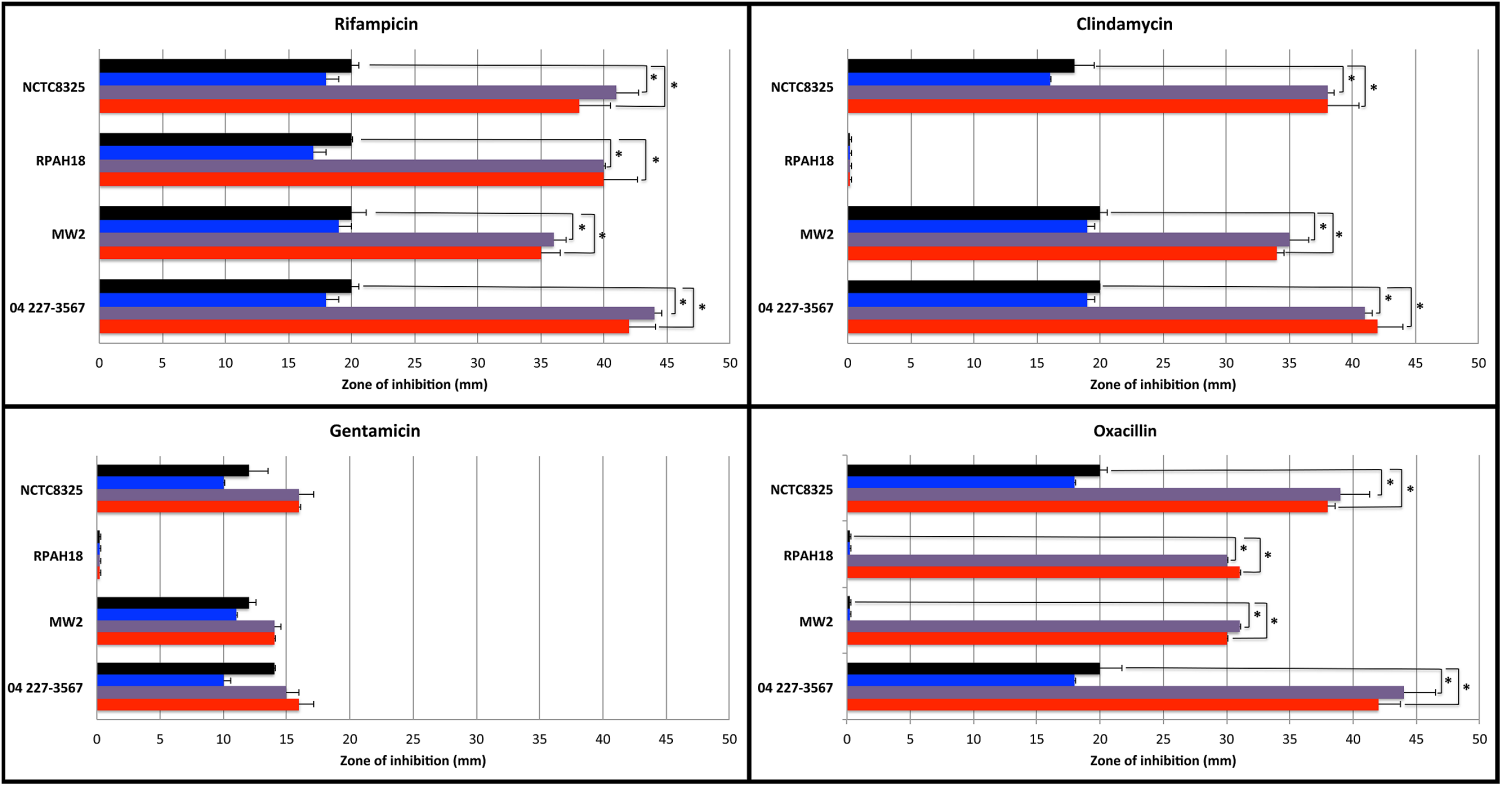
**Sensitivity of *Staphylococcus aureus* strains to antibiotics used alone and in combination with Manuka-type honeys assessed by agar disk diffusion assay.** Diameter (in mm) of zones of inhibition around 4 μg-impregnated antibiotic disks on TSA plates without honey (black bar), and in presence of 5% sugar solution (blue bar), 5% manuka honey (purple bar), or 5% Medihoney (red bar). Mean values are presented and error bars indicate one SD. Asterisks above the honey-antibiotic combination treatments indicate statistically significant differences from the antibiotic treatment alone, as determined by ANOVA analysis with a *post hoc* Dunnett’s test. Data for rifampicin (top left) is copied with permission from Müller et al. (2013).

Agar diffusion tests were performed to visualize the possible interactions between manuka honey or Medihoney and each antibiotic against each *S. aureus* strain (**Figure 1**). Sensitivity was measured based on the diameter of the zone of growth inhibition for antibiotics alone, or in combination with 5% (sub-MIC) manuka honey or Medihoney. A sugar solution made to be isotonic with honey was also used at 5% to examine the sole effect of sugar in combination with antibiotics. Additive effects are indicated by an increase in diameter of the inhibition zone with both antibiotics and honey compared to the use of an antibiotic alone.

For rifampicin, both MRSA and non-MRSA strains were sensitive and showed additive effects with the addition of 5% Medihoney or manuka honey, where inhibition zones were approximately doubled, consistent with our previous report (Müller et al., 2013). A similar effect was observed for oxacillin, where sensitive strains showed an approximate doubling of the inhibition zone with the addition of either honey, and resistant strains became sensitive with ∼30 mm zones of inhibition. This agrees with a recent report where oxacillin and manuka honey restored oxacillin susceptibility to a MRSA strain (Jenkins and Cooper, 2012b). Clindamycin and honey gave an additive (approximately double) activity for all strains, except for MRSA strain RPAH18, which remained resistant. In contrast, gentamicin produced little to no additive effects with either honey for all *S. aureus* strains. This agrees with a recent report where gentamicin and manuka honey combinations had no effect against an epidemic MRSA strain (EMRSA-15; Jenkins and Cooper, 2012a).

Sugar is considered to be an important antibacterial component of honey (Allen et al., 1991), therefore possible additive effects of sugar alone were also tested. None of the tested antibiotics in combination with sugar (at equivalent concentrations to honey used) showed any increase in the zones of inhibition of growth for any of the tested strains (**Figure 1**), suggesting that the sugar content of honey in these assays is unlikely to be responsible for the additive effects observed.

### HONEY AND ANTIBIOTICS SYNERGISTICALLY INHIBIT *S. aureus* PLANKTONIC GROWTH AND BIOFILM FORMATION, BUT THIS VARIES WITH THE ANTIBIOTIC AND STRAIN

The results above indicate that manuka honey interacts positively with most antibiotics to inhibit *S. aureus* growth. To test whether these effects were synergistic, as opposed to just additive, checkerboard microdilution assays were performed. Both planktonic growth and the degree of biofilm formation on an abiotic surface were tested with various concentrations of each honey and each antibiotic alone, and in all possible pairwise combinations (**Table 2**). For all *S. aureus* strains the type of effect, including additive and synergistic effects for antimicrobial agents alone and in combination with honey, was identical for both planktonic growth and biofilm formation. **Table 2** summarizes the MBIC obtained from crystal violet checkerboard assays for the four *S. aureus* strains along with the corresponding FICI. No antagonism (FICI > 4) was observed with any combination.

**Table 2:** Checkerboard analysis of combined effects of honey and antibiotics on *S. aureus* growth and biofilm formation.

	Antibacterial agent		Planktonic growth inhibition						Biofilm prevention					
			Antibiotic MIC (μg/mL)		Honey MIC (μg/mL)		FICI	Synergy (<0.5)	Antibiotic MBIC (μg/mL)		Honeys MBIC (μg/mL)		FICI	Synergy (<0.5)
	Antibiotic^1^	Honey	Alone	Combined with honey	Alone	Combined with antibiotic			Alone	Combined with honey	Alone	Combined with antibiotic		
NCTC8325	Rifampicin	Medihoney	0.039	0.0024	8	3	0.445	✓	0.039	0.0024	8	3	0.445	✓
	Rifampicin	Manuka	0.039	0.0024	8	3	0.445	✓	0.039	0.0024	8	3	0.445	✓
	Clindamycin	Medihoney	0.3	0.01	8	3	0.405	✓	0.3	0.01	8	3	0.405	✓
	Clindamycin	Manuka	0.3	0.01	8	3	0.405	✓	0.3	0.01	8	3	0.405	✓
	Gentamicin	Medihoney	0.6	0.2	8	7	1.195		0.6	0.2	8	7	1.195	
	Gentamicin	Manuka	0.6	0.002	8	7	0.8782		0.6	0.0024	8	7	0.8782	
	Oxacillin	Medihoney	0.25	0.008	8	3	0.405	✓	0.25	0.008	8	3	0.405	✓
	Oxacillin	Manuka	0.25	0.008	8	3	0.405	✓	0.25	0.008	8	3	0.405	✓
MW2	Rifampicin	Medihoney	0.039	0.0024	8	3	0.435	✓	0.039	0.0024	8	3	0.435	✓
	Rifampicin	Manuka	0.039	0.0024	8	3	0.435	✓	0.039	0.0024	8	3	0.435	✓
	Clindamycin	Medihoney	0.3	0.01	8	3	0.405	✓	0.3	0.01	8	3	0.405	✓
	Clindamycin	Manuka	0.3	0.03	8	3	0.475	✓	0.3	0.01	8	3	0.475	✓
	Gentamicin	Medihoney	0.625	0.2	8	5	0.945		0.625	0.2	8	5	0.945	
	Gentamicin	Manuka	0.625	0.2	8	6	1.07		0.625	0.2	8	6	1.07	
	Oxacillin	Medihoney	>20	0.06(S)^2^	8	6	0.753		>20	0.06	8	6	0.753	
	Oxacillin	Manuka	>20	0.06(S)	8	6	0.753		>20	0.06	8	6	0.753	
04-227-3567	Rifampicin	Medihoney	0.039	0.0024	8	3	0.445	✓	0.039	0.0024	8	2	0.32	✓
	Rifampicin	Manuka	0.039	0.0024	8	3	0.445	✓	0.039	0.0024	8	2	0.32	✓
	Clindamycin	Medihoney	0.3	0.01	8	3	0.405	✓	0.3	0.01	8	3	0.405	✓
	Clindamycin	Manuka	0.3	0.01	8	3	0.405	✓	0.3	0.01	8	3	0.405	✓
	Gentamicin	Medihoney	0.5	0.16	8	5	0.955		0.5	0.16	8	5	0.945	
	Gentamicin	Manuka	0.5	0.16	8	5	0.955		0.5	0.16	8	5	0.945	
	Oxacillin	Medihoney	0.25	0.008	8	3	0.407	✓	0.25	0.008	8	3	0.407	✓
	Oxacillin	Manuka	0.25	0.008	8	3	0.407	✓	0.25	0.008	8	3	0.407	✓
RPAH18	Rifampicin	Medihoney	0.078	0.0024	8	3	0.405	✓	0.078	0.0024	8	3	0.36	✓
	Rifampicin	Manuka	0.078	0.0024	8	3	0.405	✓	0.078	0.0024	8	3	0.36	✓
	Clindamycin	Medihoney	>20	>20	8	8	2		>20	>20	8	8	2	
	Clindamycin	Manuka	>20	>20	8	8	2		>20	>20	8	8	2	
	Gentamicin	Medihoney	>20	>20	8	8	2		>20	>20	8	8	2	
	Gentamicin	Manuka	>20	>20	8	8	2		>20	>20	8	8	2	
	Oxacillin	Medihoney	>20	0.06(S)	8	7	0.8782		>20	>20	8	7	0.7832	
	Oxacillin	Manuka	>20	0.06(S)	8	6	0.8782		>20	0.06	8	6	0.6732	

*^1^Data for rifampicin is copied with permission from Müller et al. (2013)*.

*^2^S = clinically susceptible to oxacillin based on EUCAST breakpoints (EUCAST, 2014) for *S. aureus* (S < 2 μg/mL)*.

Manuka honey and Medihoney each had an MBIC of 8% (w/v) for all of the *S. aureus* strains tested, including NCTC8325 and both the MRSA (RPAH18, MW2) and non-MRSA (042273567) clinical isolates (**Table 2**), consistent with our previous data (Lu et al., 2014). Results for rifampicin were similar to those observed in the agar diffusion assays, where all strains showed increased sensitivity to rifampicin and honey when used in combination compared to the single treatments alone, and this interaction was found to be synergistic (FICI < 0.5). For strains that were already sensitive to oxacillin (non-MRSA strains NCTC8325 and 042-227-3567), synergistic effects were observed between oxacillin and both honeys. Although resistant strains (MRSA strains, RPAH18, and MW2) did not show mathematically synergistic effects with the oxacillin and honey combinations, both strains went from being clinically resistant (MIC > 20 μg/mL) to sensitive (MIC < 0.06 μg/mL) to oxacillin (based on EUCAST susceptibility breakpoints; EUCAST, 2014) with the addition of honey. MW2 became sensitive in combination with both manuka and Medihoney, while RPAH18 showed sensitivity only in combination with manuka honey (**Table 2**).

Combinations of clindamycin with manuka and Medihoney were synergistic against all *S. aureus* strains except MRSA strain, RPAH18, which remained resistant. No synergistic effects were observed with gentamicin, however, strains displayed increased susceptibility to the combination of this antibiotic with either honey, again with the exception of MRSA strain RPAH18, which remained resistant (MIC > 20 μg/mL) to gentamicin.

## DISCUSSION

To address the urgent problem of antibiotic resistance, this study evaluated the antimicrobial activity of combinations of NZ manuka-type honeys with four antibiotics commonly used to treat *S. aureus-*related skin infections. We demonstrate increased sensitivity to both antibiotics and manuka-type honeys when they are used in combination, even when strains are clinically resistant to a particular antibiotic; however, this depends on the antibiotic and on the *S. aureus* strain. For all tested *S. aureus* strains, the rifampicin-honey combination is the most promising, with synergistic inhibition observed for both planktonic growth and biofilm formation. Thus our work suggests that NZ manuka-type honey has excellent potential as an alternative natural antimicrobial agent for use in combination therapy with rifampicin against *S. aureus*-related skin infections.

Although quantifiable synergism, as measured by the FICI, was not observed for every honey-antibiotic combinations tested in this study, increased sensitivity was observed for most combinations. For example, the MIC and MBIC of oxacillin, clindamycin, and gentamycin decreased from three- to eightfold, when used in combination with manuka-type honeys. Additionally, while the MRSA strains were resistant to oxacillin beyond clinically achievable concentrations (>20 μg/mL), when combined with the NZ manuka-type honeys, susceptibility was reduced to within clinically achievable peak plasma concentrations (e.g., <63 μg/mL; Amsden, 2009). This overall, and at times dramatic, improvement in antibiotic sensitivity when combined with honey suggests the excellent potential of the use of manuka-type honeys as an antibiotic adjuvant in combinational therapies to treat antibiotic-resistant chronic wound infections.

Synergistic effects between oxacillin and manuka honey against MRSA growth has been reported (Jenkins and Cooper, 2012b). This was proposed to be due to the corresponding decreased transcription of the MRSA-specific penicillin binding protein (PBP2A) that has markedly reduced affinity to β-lactams compared to endogenous *S. aureus* PBP enzymes (de Lencastre et al., 2007; Llarrull et al., 2009). Our results show, however, that negative regulation of PBP2A by manuka honey is unlikely to be the sole mechanism responsible for the synergistic effect with β-lactams, as strong synergism was also detected against non-MRSA strains, which do not have the *mecA* gene (Sturenburg, 2009).

One of the ways in which drug combinations work is when both drugs act on sequential or orthogonal steps of an essential physiological pathway, achieving ‘a like plus like’ effect (Kalan and Wright, 2011). Clindamycin and gentamicin inhibit bacterial cell growth by targeting the 50S and the 30S subunits of the ribosome, respectively (Schlunzen et al., 2001). Since NZ manuka-type honey alters the levels of protein synthesis components, including ribosomal proteins (Blair et al., 2009; Packer et al., 2012), the synergistic effect of honey in combination with either of these antibiotics may be due to this ‘like plus like’ effect on the protein synthesis pathway, shutting it down more effectively.

Bactericidal drugs such as β-lactams (e.g., oxacillin) and aminoglycosides (e.g., gentamicin) contribute to cell death by stimulating hydroxyl radical formation via the Fenton reaction (Kohanski et al., 2007). A major antibacterial component of honey, including manuka, is hydrogen peroxide (White et al., 1963) and it acts via the production of hydroxyl radicals via the Fenton reaction (Brudzynski and Lannigan, 2012). The enhanced sensitivity of *S. aureus* to the bactericidal antibiotics upon addition of honey could be at least partly due to an increase in hydroxyl radical production. This might also explain why honey-oxacillin synergy is observed with non-MRSA as well as MRSA strains; it is due to enhanced hydroxyl radical production rather than, or in addition to, any effect on PBP2A. However, we suspect other factors in honey are responsible for the synergy because peroxide activity in manuka-type honeys is not essential for the complete inhibition of bacterial growth (Lu et al., 2013).

Interestingly, no increase in antibiotic susceptibility against clindamycin and gentamicin was found with the clinical MRSA isolate, RPAH18. The reason for this is unclear, but it may be due to a different response by this strain toward the stresses induced by the antibiotics and/or honey such as additional eﬄux systems or other as yet uncharacterized physiological barriers that prevent the entry, accumulation or action of these antibiotics. This response by RPAH18 also indicates that the mechanism of honey-antibiotic synergy is distinct from its mode of growth inhibition, as all *S. aureus* strains tested to date, including many different MRSA strains, are inhibited by honey to a very similar extent (Blair and Carter, 2005). The implication of this strain variability is that although honey may represent a good antibiotic adjuvant, it may not always work. Dissection of the synergistic mechanism between honey and antibiotics is necessary to understand this strain variation. However, it should be noted that manuka-type honeys remained effective at inhibiting growth and biofilm formation of RPAH18 when used alone at a slightly higher, easily achievable concentration (>8%).

While honey may work well on its own to effectively treat chronic wounds, our data also support the use of honey dressings in situations where an additional systemic antibiotic is desirable, for example in certain patients vulnerable to sepsis. Synergism and improved sensitivity with combination treatments were obtained with a low concentration (<8%) of manuka-type honey. This is easily achieved since wound gel and honey dressings typically contain >90% honey, and would still be effective after significant dilution with wound exudate. The Medihoney used in this study is an existing commercially available and FDA-approved wound dressing. As processed manuka honey, including gamma sterilization, Medihoney displayed the same level of effectiveness as unprocessed manuka honey in preventing the growth and biofilm formation of *S. aureus*. Controlled clinical trials could therefore be used to examine the value of manuka-based honey dressings both alone or in combination with antibiotics in the effective treatment of recalcitrant wounds. This is particularly promising as very few new antibiotics are being developed, and the antibiotic resistance problem is increasing, often with resistance developing in chronic infected wounds prior to completion of therapy. In addition, topical treatment of skin and chronic wound infections with honey would target areas of the wound bed that a systemically administered antibiotic may not be able to enter, such as the necrotic wound tissue. The overall effectiveness of NZ manuka-type honeys and antibiotic combinations could represent a new model of treatment for wound-associated infections, where the antibiotic acts systemically entering from the bottom of the wound bed (as well as functioning as a prophylactic for systemic infections), while honey acts topically from the top of the wound. This approach represents an immediate practical solution for the treatment of difficult to treat *S. aureus*-related infections.

## Conflict of Interest Statement

Ralf Schlothauer is an employee of Comvita New Zealand Limited which trades in medical grade manuka honey (Medihoney). Comvita New Zealand Limited provided partial funding and materials for the work described in the manuscript. The other authors declare that the research was conducted in the absence of any commercial or financial relationships that could be construed as a potential conflict of interest.
